# Effect of Heat Stress on the Biosynthesis of Exopolysaccharides from *Rhodotorula glutinis* YM25079 and Its Underlying Mechanisms

**DOI:** 10.3390/jof11120883

**Published:** 2025-12-14

**Authors:** Rong Huang, Minrao Lu, Caina Guo, Taishen Wang, Jingdie Fan, Chengmei Zhang, Jingwen Qiu, Yuan Chen, Qi Zhang

**Affiliations:** Faculty of Life Science and Technology, Kunming University of Science and Technology, Kunming 650500, China; hunagrong123@163.com (R.H.); joyous0519@163.com (M.L.); 18468150409@163.com (C.G.); wt202506@163.com (T.W.); fanl001@outlook.com (J.F.); chrnmei@163.com (C.Z.); jiqiu@kust.edu.cn (J.Q.)

**Keywords:** *Rhodotorula glutinis*, exopolysaccharide biosynthesis, heat stress, structure, transcriptome sequencing

## Abstract

In this study, the effect of heat stress on the synthesis and the structural and physicochemical properties of exopolysaccharides (EPSs) from *Rhodotorula glutinis* YM25079 as well as its underlying mechanisms were explored. The results showed that the monosaccharide compositions of the purified YM25079 EPSs produced under normal culture conditions and heat stress (named EPS Y-1 and EPS Y-2, respectively) were consistent. Analyses of ion-exchanged chromatography, Fourier transform infrared spectroscopy and nuclear magnetic resonance spectroscopy suggested that these two EPSs should be dextran, consisting mainly of α-(1→6)-linked glucopyranose units with α-(1→3) branches. Scanning Electron Microscope observed obvious differences in their surface morphologies, with EPS Y-1 showing a smooth, glossy lamellar structure and EPS Y-2 showing an irregular porous structure. According to Atomic Force Microscopy analysis, they formed aggregations with different cohesive structures. EPS Y-2 also had higher molecular weight and thermal stability than EPS Y-1, while EPS Y-1 had better α-amylase inhibitory activity. In addition, transcriptomic analysis unveiled changes in the metabolic pathways related to the uptake and utilization of carbon, nitrogen and phosphor sources, the biosynthesis of steroid and the oxidoreductase activity, as well as the regulatory genes implicated in the EPS biosynthesis under heat stress.

## 1. Introduction

A variety of environmental stresses are present in the natural environment such as temperature, osmotic pressure and pH changes as well as nutrient starvation, oxidation, drought, salt, heavy metals and UV radiation, etc. [1]. Environmental stress can cause changes in intracellular metabolic pathways, inhibition of enzyme activities, destruction of cellular structures and impaired transport of substances across membranes, which are detrimental to cell growth. To optimize growth and productivity under adverse environmental conditions, microorganisms have developed diverse regulatory mechanisms to sense and respond to external pressures [2]. Production of extracellular polymeric substances, primarily composed of EPSs, has been documented as a stress response and survival mechanism for many microorganisms [3,4,5,6]. For example, EPS yield of *Rhodotorula* sp. strain CAH2 increases constantly with increasing concentrations of Al, NaCl and PEG-6000 to survive under abiotic stresses [6].

Microbial EPS is a kind of polymer compound formed by many of the same or different monosaccharides via different sequences of glycosidic bonds with different types of branching, which is intracellularly synthesized and secreted outside in the process of growth and metabolism [7]. In addition, yeast EPS has a variety of physiological activities [8,9], which have become one of the research hotspots in recent years. Several studies have shown that factors such as molecular weight, monosaccharide composition, glycosidic bonding, functional groups and surface morphology of microbial EPS affect the functional properties of EPS [10,11,12]. Among them, the molecular weight of EPS has an important influence on its biological activity. Typically, larger molecular weight EPS is positively correlated with antitumor activity compared to EPS with lower molecular weight over a range of concentrations [13]. Surface morphology of microbial EPS is closely related to the adsorption, hydrophilicity and emulsification of EPS [9]. In addition, the glycosidic bond is an important structural unit in the EPS molecule and has a significant impact on the properties of EPS. High content, multiple types and specific spatial configurations of glycosidic bonds modulate the solubility, stability and hydrophilicity of EPS [14]. Due to the complex structure of EPS, the study of the conformational relationship between structure and activity is not sufficiently deep at present, especially for the changes in the structure and functional adaptability of EPS under environmental stresses.

Changes in environmental conditions may affect the biosynthetic pathways of microbial EPS, thereby altering their structure and biological activity. It has been found that under salt stress conditions, different strains exhibited elevated EPS, altered monosaccharide composition, as well as superior physicochemical properties [15,16]. Under cadmium stress, *Enterococcus faecalis* CX2-6 was found to increase the ability to secrete EPS with enhanced adsorption capacity [17]. These studies suggest that antagonism occurs in strains under stress conditions through the secretion of EPS with complex structures and regulatory activities. Currently, a number of researchers are devoted to the discovery of the relationship between bacterial EPS synthesis and adaptation to environmental stress [18,19]. Song [18] showed that *Chlorella*-Arca cells used EPS rather than lipids as important energy storage in response to high temperature stress and secreted EPS into the environment to maintain intracellular osmotic pressure homeostasis. Studies by Yang [19] showed that the EPS production and environmental adaptation of Rhizobia were positively regulated by the genes *iscS*, *envZ* and *asnC*. However, studies on the mechanisms related to how environmental stress affects EPS synthesis in yeast have rarely been reported [6].

*Rhodotorula glutinis* (*R. glutinis*) is a promising aerobic microorganism that synthesizes many valuable secondary metabolites such as lipids [20], carotenoids [21] and EPSs [22]. With increasing global temperatures, heat stress has become a common environmental stressor worldwide. It has been shown that the synthesis of secondary metabolites of microorganisms under heat stress undergoes large changes [23]. However, whether heat stress affects the synthesis of EPS in *R. glutinis* and yeast environmental adaptation remains a topic to be explored. In this study, *R. glutinis* YM25079 was used as an experimental subject. According to our previous study [24], this strain is a low-temperature-adapted strain with an optimal growth temperature of 15 °C; it exhibited retarded growth when the temperature exceeded 30 °C. YM25079 EPSs produced under optimal growth temperature (15 °C) and heat stress (30 °C) were firstly isolated and purified. The effects of heat stress on the metabolism, structure and properties of YM25079 EPS were investigated and the influence of heat stress on the biosynthesis of EPS was also explored by combining with transcriptome analysis. This study first systematically explored the EPS biosynthetic kinetics, molecular structure–activity relationships and metabolic regulatory networks of *R. glutinis* YM25079 under stress microenvironments. We expect our results not only to promote the investigations into the mechanism underlying yeast’s responses to environmental changes, but also to provide theoretical support for developing adaptive enhanced strains based on synthetic biology strategies.

## 2. Materials and Methods

### 2.1. Yeast Strain and Cultural Conditions

*R. glutinis* YM25079 was isolated from Lugu Lake, Lijiang City, Yunnan Province, China. YM25079 was first cultured with a 5 mL YPD liquid medium (yeast powder 10 g/L, peptone 20 g/L, glucose 20 g/L, pH natural) and incubated on a shaker at 160 rpm at 15 °C for 36 h. It was transferred to 50 mL of YPD liquid medium and incubated in a shaker at 160 rpm at 15 °C until the OD_600_ was about 1.68 or so as seed solution. Then it was inoculated into 50 mL of YP liquid medium (yeast powder 10 g/L, peptone 20 g/L, pH natural) with different carbon sources at 1% inoculum volume and incubated at 15 °C and 30 °C at 160 rpm for 5 days, respectively. Three replicates were set for each sample.

### 2.2. Growth and Metabolic Characterization of Strain YM25079

#### 2.2.1. One-Way Experiments with Culture Media

YP liquid medium was used as the base medium to fix the amount of yeast powder, peptone in the medium, pH natural. The effects of carbon sources (glucose, sucrose, maltose, mannose, xylose, fructose) and carbon additions (2%, 4%, 6%, 8%) on the EPS production of *R. glutinis* YM25079 under heat stress (30 °C) versus optimal temperature (15 °C) were investigated, respectively. According to Li’s method [25] with minor modifications, the phenol-sulfuric acid method was performed to determine EPS content. Briefly, 0.4 mL of phenol solution (50 g/L) was added to 0.2 mL of sample solution. After thorough mixing, 2 mL of concentrated sulfuric was added into the tube rapidly. After standing for 5 min, the mixture was placed in a boiling water bath for 25 min. The absorbance at OD_490_ was measured after cooling.

#### 2.2.2. Growth and Metabolic Profiling

The optimized medium was used as the base sugar production medium, and fermentation was carried out at 30 °C and 15 °C in a shaker at 160 rpm with a loading volume of 50 mL. The OD_600_ and EPS production of the strains were determined at different time points (6 h, 12 h, 24 h, 36 h, 48 h, 72 h, 96 h, 120 h), and the growth and metabolic curves were plotted.

#### 2.2.3. Kinetic Modeling of Strain Growth [26]

The strain growth kinetics of YM25079 was modeled using the logistic equation, which was as follows:

(1) $$\frac{dX}{dt}=\;\mu_{max}(1\;-\;\frac{X}{X_{max}})X$$

(X is the cell mass concentration g·L^−1^; μ is the specific growth rate h^−1^; and t denotes the time h. The biomass as a function of time can then be obtained by integrating Equation (1) and substituting in X = X_0_).

(2) $$X(t)=\frac{X_{0}e^{\mu_{max}t}}{1-\frac{X_{0}}{X_{max}}(1-e^{\mu_{max}t})}$$

(The maximum specific growth rate (μ_max_), X_0_ and X_max_ were obtained by nonlinearly fitting X(t) to time t. The correlation coefficient (R^2^) evaluates the degree of model fit).

Modeling the EPS synthesis of YM25079 using the Luedeking–Piret equation, which was as follows:

(3) $$\frac{dP}{dt}=\;\alpha X\;+\;\beta \frac{dX}{dt}$$

(P is the product concentration, g·L^−1^; α and β are empirical constants, and β$\frac{dX}{dt}$ and αX denote the growth-associated and non-growth-associated EPS synthesis rates, respectively). Substituting Equation (2) into Equation (3), we get

(4) $$P(t)=P_{0}+\alpha X_{0}[\frac{e^{\mu_{max}t}}{1-\frac{X_{0}}{X_{max}}(1-e^{\mu_{max}t})}-1]+\beta \frac{X_{max}}{\mu_{max}}\ln[1-\frac{X_{0}}{X_{max}}\left(1-e^{\mu_{max}t})\right]$$

(Nonlinear fitting of P(t) to t yields α, β and initial P_0_, and R^2^ evaluates the degree of model fit. The EPS specific growth rate $\frac{dP/dt}{X}$ can be obtained from Equation (3)).

### 2.3. Extraction and Purification of YM25079 EPS

EPS was extracted according to Li’s method with minor modifications [25]. The fermentation liquid was centrifuged (5000 rpm/min, 6 min, 4 °C), and 2 times the volume of ice ethanol (volume fraction: 95%) was added to the supernatant and left at 4 °C for 12 h. Appropriate amount of distilled water was added to dissolve the EPS, and protein was removed by Sevag method. The deproteinized sugar solution was transferred to a dialysis bag and dialyzed using distilled water. The distilled water was changed every 6 h and dialyzed for 2–3 days, then the dialyzed sugar solution was dispensed and placed at −80 °C, followed by freeze-drying for 2–3 days. The purification of EPS was carried out by Hu’s method [27].

### 2.4. Molecular Weight and Monosaccharide Composition Analysis

According to the method of Chen [28], the molecular weight and conformation of EPS were determined using high performance liquid molecular exclusion chromatography coupled with a laser light scattering instrument and differential detector (HPSEC-MALLS-RI) tandem detection. EPS was prepared as a sample solution of 2.0 mg/mL. All solutions were passed through a 0.22 μm microporous filter membrane and then analyzed by high performance gel permeation chromatography (HP-GPC). Heavy average molecular weights (Mw) was determined. Data were collected and calculated using ASTRA 8 software [22].

The monosaccharide composition of EPS was determined by high performance liquid chromatography (HPLC) [29,30]. A total of 5 mg of EPS was dissolved in 5 mL of trifluoroacetic acid (TFA) and hydrolyzed under the condition of 110 °C for 8 h. The EPS samples were hydrolyzed by blow-drying with N_2_ to remove the excess TFA and re-dissolved by adding 300 μL of water. It was then derivatized and subjected to HPLC analysis.

### 2.5. UV and FT-IR Spectrometric Analysis

The EPSs with uniform molecular weight were prepared as a 1 mg/mL aqueous solution and distilled water was used as a blank control, and the scanning wavelength of the UV-2800 spectrophotometer scanner was set at 200–400 nm [31]. The functional groups of YM25079 EPS were determined by FT-IR [7]. YM25079 EPS and KBr were ground well in the ratio of 1:100 to a flour-like consistency and pressed into transparent sheets using a tablet press. The samples were scanned 32 times in the range of 4000–400 cm^−1^ with a resolution of 4 cm^−1^.

### 2.6. Analysis of Thermal Properties

The thermal properties of purified EPS were investigated using a thermal analyzer (Netzsch TG 209 F1, Selb, Germany) with Ar_2_ as the protective gas. A total of 4–5 mg of the EPS sample was placed in an alumina crucible. The experiments were carried out at a linear heating rate of 10 °C/min over a temperature range of 40–800 °C. Plots of weight loss and heat flow relative to temperature were obtained [32].

### 2.7. Scanning Electron Microscopy (SEM) and Atomic Force Micrograph (AFM) Analysis of EPS

The surface morphology of purified EPS was observed using scanning electron microscopy (Hitachi SU3900, Tokyo, Japan). After gold spraying, the appropriate amount of EPS powder was immobilized on the scanning electron microscope stage and observed under 2.0 KV voltage. The purified EPS was dissolved in ultrapure water to a concentration of 1 mg/mL, and the EPS was fully dissolved by stirring in a water bath at 37 °C. Then serially diluted to a final concentration of 1 μg/mL. A total of 5 μL of EPS solution was dropped directly onto the surface of freshly peeled mica flakes, dried at room temperature and used for subsequent AFM testing [33].

### 2.8. Nuclear Magnetic Resonance (NMR) Spectroscopy Analysis of EPS

The EPS powder was completely dissolved in D_2_O, configured into a sample solution of 80 mg/mL and transferred to an NMR tube. The ^1^H NMR and ^13^C NMR of EPS were determined at 600 MHz using nuclear magnetic resonance spectroscopy (Bruker, Fällanden, Switzerland). The experiments were carried out at room temperature.

### 2.9. Rheological Analysis

The EPS samples were configured into a 10 mg/mL solution. The effect of system temperature on the apparent viscosity of the EPS solutions was determined by a rheometer at temperatures of 5 °C, 25 °C and 45 °C, respectively. The independent variable was the shear rate in the range of 10^−1^–10^2^s^−1^ [34].

### 2.10. Determination of α-Amylase Inhibitory Activity

A total of 20 μL sample solution at different concentrations (2, 4, 6, 8, 10 mg/mL) was taken to be vortex mixed with 20 μL of α-amylase solution (1.0 U/mL), then heated with a water bath at 37 °C for 10 min. Subsequently, 40 μL 1.00% starch solution was added and mixed, and the reaction was continued for 10 min at 37 °C. Finally, 80 μL DNS reagent was added and the reaction was terminated by heating in a boiling water bath for 5 min, and the absorbance value was measured at wavelength 540 nm after dilution with 1 mL water. The α-amylase inhibition rate was calculated as follows:

α-Amylase inhibition rate (%) = [1 − (A − B)/C] × 100%.

### 2.11. Transcriptome Sequencing

#### 2.11.1. Culture Conditions and Cell Sample Preparation

For transcriptome sequencing, YM25079 was pre-incubated in yeast sugar production medium (1% yeast extract, 2% peptone and 6% maltose) at 15 °C for 24 h, then was cultivated at 15 and 30 °C for another 12 h, respectively, with three technical replicates collected for each sample. The culture broth was centrifuged at 5000× rpm for 5 min at 4 °C to remove the medium and collect the organisms.

#### 2.11.2. Total RNA Extraction and Library Construction

Total RNA was extracted using the TRIzol Reagent Kit (Qiagen, Hilden, Germany) following the manufacturer’s instructions. RNA integrality and concentration were assessed on the Agilent 5300 bioanalyzer (Agilent, Santa Clara, CA, USA) and the NanoDrop 2000 Spectrophotometer (Thermo Fisher Scientific, Wilmington, DE, USA). Then, the transcriptional library was constructed using the Illumina^®^ Stranded mRNA Prep with Ligation (Illumina, San Diego, CA, USA), and sequencing was performed using the NovaSeq Reagent Kit (Illumina, USA).

#### 2.11.3. Differential Expression Analysis and Gene Function Enrichment

Raw reads were cleaned by removing adapter sequences and low-quality reads using Fastp software (v0.23.2). Cleaned reads were aligned to the assembled genome sequences of *R. glutinis* YM25079 to generate bam results using HISAT2 (v2.2.1). Samtools (v1.17) was used to sort and index these bam files, which were then assembled by StringTie (v2.2.1) to obtain unigenes and quantify their expressions. The unigenes were annotated by aligning to public databases including the NCBI non-redundant protein (Nr) database (http://www.ncbi.nlm.nih.gov), the Swiss-Prot protein database (version 2021.6) (http://www.expasy.ch/sprot, accessed on 14 October 2024), the protein families database Pfam (http://pfam.xfam.org/), the EggNOG database (version 2020.06) (http://eggnog6.embl.de), the Gene Ontology (GO) resource (https://geneontology.org/) and the Kyoto Encyclopedia of Genes and Genomes (KEGG) database (http://www.genome.jp/kegg). Differentially expressed genes (DEGs) were identified using the DESeq2 package (v1.38.3), based on an absolute value of the log_2_ (fold change) ≥ 1 and *p* < 0.05. These DEGs were subjected to GO and KEGG enrichment analyses using the clusterProfiler R package (v4.6.2). For data visualization, built-in R functions or packages ggplot2 (v3.5.1), pheatmap (v1.0.12), GOplot (v1.0.2), and ggpubr (v0.6.0) were used.

#### 2.11.4. Real-Time Quantitative PCR Analysis

To verify the reliability of the transcriptome data (BioProject ID:PRJNA1197129), 10 genes of interest were selected to test for differential expression ploidies by real-time quantitative PCR (RT-qPCR). The RT-qPCR primers were designed using NCBI primer-BLAST (https://www.ncbi.nlm.nih.gov/tools/primer-blast/, accessed on 14 October 2024), with reference to the SYBR^®^ Green fluorescent inlay method and primers synthesis was performed by Shanghai Shenggong Bioengineering Co., Ltd. (Shanghai, China). The total RNA in the samples was extracted by E.Z.N.A ^®^ Fungal RNA Kit (R6840-01, Omega Bio-tek, Norcross, GA, USA) and reversed to cDNA by the HiScript II 1st Strand cDNA Synthesis Kit (+gDNA wiper) (R212-01, Vazyme, Nanjing, China). The cDNA was used as a template for real-time fluorescence quantitative PCR analysis to compare the differences in gene expression. The RT-qPCR primers used in this study are shown in Table S6.

### 2.12. Statistical Analysis

All data was plotted in the software Graphpad Prism 8.0 (Graphpad software Inc., San Diego, CA, USA) or Origin 2022 (OriginLab software Inc., Northampton, MA, USA). Significant differences between samples were assessed using *t*-tests.

## 3. Results

### 3.1. Effect of Carbon Source and Its Additive Amount on the Yield of EPS

**Figure 1:**
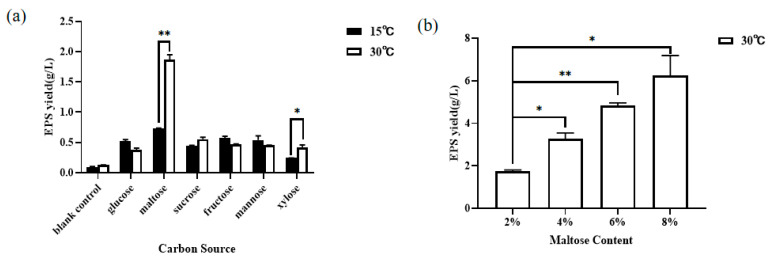
Effect of carbon source type (**a**) and maltose additive amount (**b**) on the EPS production of the *R. glutinis* YM25079. Asterisks indicate significant level: * *p* < 0.05; ** *p* < 0.01.

As shown in Figure 1a, the highest EPS production was 1.86 ± 0.12 g/L at 30 °C, which was 2.54 times higher than 0.74 ± 0.00 g/L, obtained under the normal culture condition of 15 °C, when maltose was used as carbon source. This indicated that maltose could significantly promote the biosynthesis of YM25079 EPS under heat stress. As can be seen from Figure 1b, the crude EPS yield of YM25079 increased from 1.74 ± 0.10 g/L to 6.24 ± 1.34 g/L with the increase in maltose addition from 2 to 8%. However, as the concentration of maltose increased, the increase in EPS yield gradually slowed down. In the study, the maltose addition of 6% was selected as a representative amount to ensure sufficient EPS yield for subsequent studies.

### 3.2. Growth Curves of R. glutinis YM25079 and Metabolism of EPS

**Figure 2:**
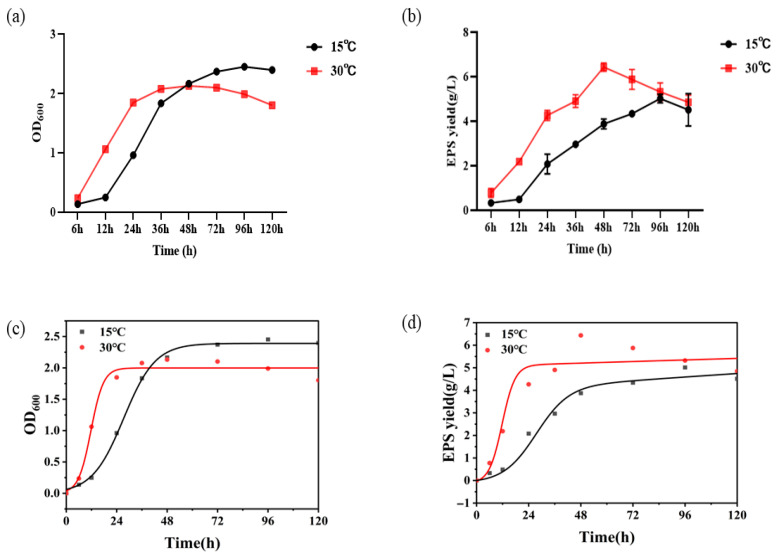
Growth and metabolic profiling of the *R. glutinis* YM25079 at different temperatures (**a**,**b**); Kinetic modeling of strain growth (**c**,**d**).

The growth of the *R. glutinis* YM25079 at different temperatures is shown in Figure 2. YM25079 grew rapidly in the first 48 h phase under heat stress. During this period, the cell metabolism was more active, accumulating abundant materials and capacity for division and value-added. Therefore, it could be considered as the most sensitive period for *R. glutinis* cells exposed to the adverse external environment. While the growth of *R. glutinis* was significantly inhibited under heat stress conditions after 48 h, demonstrating that the high energy consumption required by the *R. glutinis* cells to defend the heat stress resulted in an inability to accumulate excess energy for growth, which in turn caused a decline in biomass.

### 3.3. Kinetic Modeling Fitting of Strain Growth

Logistic and Luedeking–Piret equations were used to establish the changes in growth and product synthesis during the synthesis of EPS by YM25079, respectively. The reference values of each model are shown in Table S1, and the fitted R^2^ of the model for each experimental group was greater than 0.9. As illustrated in Figure 2c,d, both models fit the experimental values well, which can be used to characterize the YM25079 growth dynamics. As shown in Table S1, the maximum specific growth rate μ_max_ of the *R. glutinis* YM25079 under heat stress was about 0.33528 h^−1^. In contrast, μ_max_ was about 0.13286 h^−1^ under normal culture conditions.

In terms of product synthesis, YM25079 obtained a maximum EPS specific production rate of 0.87670 h^−1^ under heat stress. Neither the product formation parameter α, representing the product formation parameter associated with strain growth, nor the product formation parameter β, representing the product formation parameter not associated with strain growth, were 0, indicating that the EPS synthesis of YM25079 was partially of the growth-coupled type.

### 3.4. Purification of Crude EPSs

Crude EPSs were purified by chromatography on a DEAE-52 ion-exchange column, and the elution profiles are shown in Figure S1. Among of them, EPS-1s separated from normal culture conditions and heat stress were washed out with deionized water, which were neutral and used as purified EPSs for the following study (named EPS Y-1 and EPS Y-2, respectively). The sugar content was also found to be about 60% by the phenol sulfate method. Due to the low sugar content, EPS-2 and EPS-3 were not studied further.

### 3.5. Molecular Mass and Monosaccharide Composition of EPS

**Figure 3:**
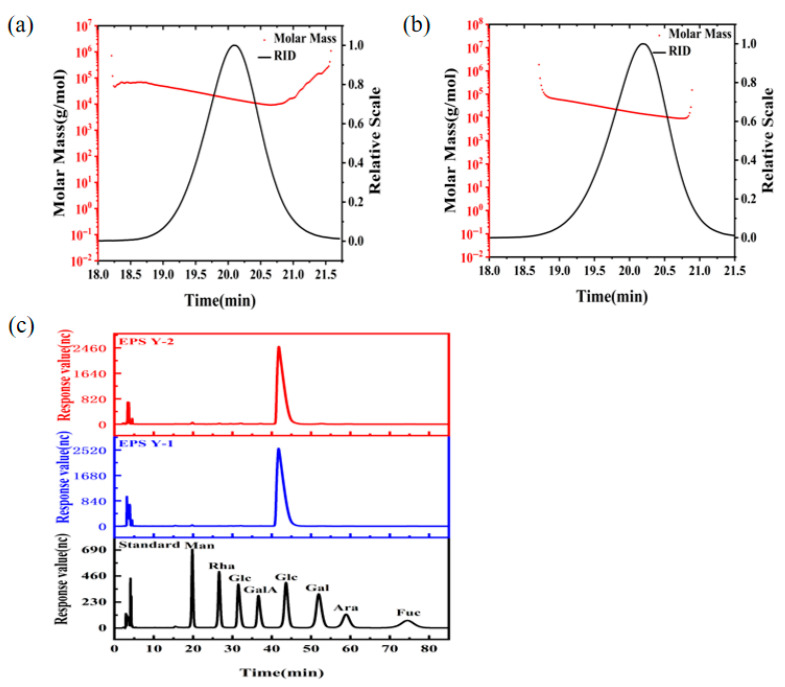
Molecular mass analysis of EPS Y-1 (**a**) and EPS Y-2 (**b**). Monosaccharide composition analysis (**c**) of EPS Y-1 and EPS Y-2.

The molecular weight of YM25079 EPS was determined by the HPGPC method. EPS Y-1 (Figure 3a) and EPS Y-2 (Figure 3b) showed a single symmetrical peak in the GPC chromatogram, indicating that both were homogeneous polysaccharides, which can be further used for the determination of the monosaccharide constituents. The heavy average molecular weights (Mw) of EPS Y-1 and EPS Y-2 were determined to be 1.844 × 10^4^ g/mol and 2.048 × 10^4^ g/mol, respectively, using the system analysis software. The slopes of the molecular conformation diagrams of EPS Y-1 and EPS Y-2 were 0.83 ± 0.05 and 0.47 ± 0.03, respectively, indicating that EPS Y-1 was rigid rod-shaped and EPS Y-2 tended to be spherical. In addition, both EPS Y-1 and EPS Y-2 were glucans (Figure 3c). The above results showed that the molecular weight of exopolysaccharide produced by strain YM25079 under heat stress conditions was higher than that of exopolysaccharide produced under normal culture conditions. At the same time, there were significant differences in the molecular configurations between the two.

### 3.6. UV and FT-IR Spectrum Observation

From Figure S2a, the purified EPS Y-1 and EPS Y-2 did not show significant absorption peaks at 260 nm and 280 nm, indicating that they did not contain nucleic acids and proteins [35]. FT-IR analysis was further carried out to characterize the molecular movement of EPS produced by strain YM25079 with or without heat stress. As shown in Figure S2b, the infrared spectra with and without heat stress were very similar, and both had the characteristic absorption peaks of saccharides [36]. The peaks at 1730–1700 cm^−1^ indicated the absence or minimal content of uronic acid [37], which was consistent with the result of the monosaccharide composition analysis. Absorption peaks in the range of 1200–1000 cm^−1^ indicated the presence of C-O-C and C-O-H stretching vibrations of the pyranose ring [38]. The peak at 927 cm^−1^, on the other hand, suggested the presence of an α-configuration glycosidic bond in the structure of the EPS, which was also verified to be related to the presence of the pyranose ring [39]. The peak at 1544 cm^−1^ indicated the absence of proteins, which was consistent with the result of the UV spectroscopy [40].

### 3.7. Thermal Analysis

**Figure 4:**
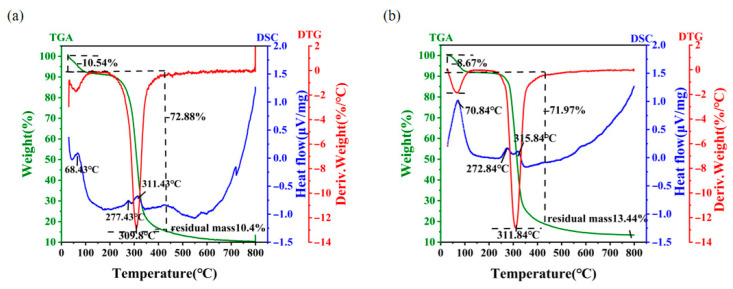
Thermogravimetry and DSC thermogram of EPS Y-1 (**a**) and EPS Y-2 (**b**).

Stabilization of EPS at elevated temperatures is an important factor in the maintenance of their biological function [41]. Thus, the industrial and commercial utilization of EPS is closely related to its thermodynamic properties [42]. Figure 4 demonstrates the results of thermogravimetric analysis (TGA), with three stages of thermal weight loss for both EPS Y-1 (Figure 4a) and EPS Y-2 (Figure 4b). In the first stage, a mass loss of approximately 10% was observed at a similar temperature range between 30 °C and 120 °C for the two EPSs, which may be caused by the evaporation of water. The second stage was the rapid thermal decomposition of EPS, which could be attributed to the destruction of its C-C and C-O bonds. In this stage, dramatic mass losses of 72.88% from 120 °C to 370 °C for EPS-Y1 and 71.97% from 120 °C to 405 °C for EPS-Y2 were observed. Thereafter, the thermal weight loss was gradually reduced to constant weight, with 10.4% residual mass for EPS-Y1 and 13.44% residual mass for EPS-Y2 around 800 °C, suggesting that EPS-Y2 should possess relatively high heat resistance properties. Additionally, the differential thermogravimetric (DTG) mass loss spectrum revealed the degradation temperature for EPS-Y1 and EPS-Y2 was ca. 309 °C, which was higher than that of EPS from *Lactobacillus plantarum* KF5 [43]. Differential scanning calorimeter (DSC) analysis was conducted to show endothermic and exothermic processes with the rising temperature. Three different endothermic peaks were observed at 68.43 °C, 277.43 °C, 311.43 °C of EPS Y-1 and 70.84 °C, 272.84 °C, 315.84 °C of EPS Y-2, which were corresponding to pyrolysis processes during the first two stages shown in the TGA results. All these results showed that YM25079 EPS was a kind of stable macromolecular compound under heat stress, which can be used to make high-temperature resistant biomaterials.

### 3.8. SEM Analysis of EPS

**Figure 5:**
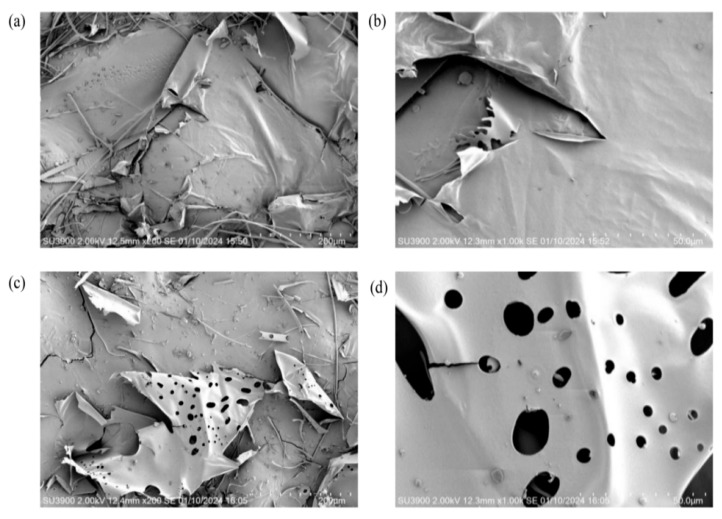
SEM showing the microscopic features at ×200 (left) and ×1000 (right) magnification of EPS Y-1 (**a**,**b**) and EPS Y-2 (**c**,**d**).

**Figure 6:**
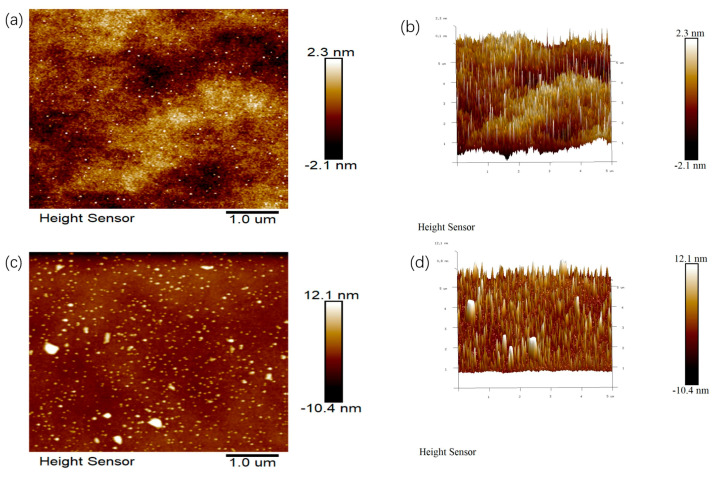
AFM images showing the planar (left), cubic view (right) of EPS Y-1 (**a**,**b**) and EPS Y-2 (**c**,**d**).

SEM is commonly used to explore the surface morphology of macromolecular materials, which can reveal certain physical and chemical properties. In our study, the surface morphology of EPS Y-1 (Figure 5a,b) was characterized by smooth, glossy flakes. Some of the lamellae had some curling, indicating a certain degree of flexibility, which gave them the potential to be made into plasticized membrane materials [43]. In contrast, EPS Y-2 (Figure 6c,d) had an irregular porous structure.

### 3.9. AFM Analysis of EPS

AFM was used to compare the surface morphology and chain conformation of EPS Y-1 and EPS Y-2 by detecting the interatomic interaction forces between the sample surface and the probe. As shown in Figure 6, their molecular chains agglomerated into spherical particles in the aqueous solution to support its branched structure, and the size and height of the agglomerates were inconsistent, which was related to the strength of aggregation of the EPS molecules [44]. Comparison of the morphology of these two EPSs revealed that the EPS molecules polymerized more under heat stress while EPS Y-1 was smoother in terms of surface roughness, which was consistent with the SEM results. This polymerization of EPS molecules occurs due to the strong interactions of hydroxyl groups within and between molecules [45]. Consequently, these morphological features may affect the biological activities and functional properties of YM25079 dextran.

### 3.10. NMR Spectroscopy Analysis

As displayed in Figure S3, the chemical shifts in this sample were mainly concentrated between δ 3.0–5.5 ppm. The strong peak at 4.79 ppm was the absorption peak produced by D_2_O. There was a signal peak at 4.88 ppm, indicating that the EPS was linked by α-(1,6) glycosidic bonds [46]. In addition, a signal peak at 5.34 ppm could be assigned to the α-(1,3) connection [47].

As shown in Figure S4, the carbon spectrum signal of this sample was mainly concentrated between δ 95–100 ppm. The absence of absorption peaks between 101 and 105 ppm indicated that the glycosidic bond type of EPS was α-configuration [13,47]. The absence of signal at 160–180 ppm indicated that it did not contain glyoxalate, which was consistent with the results of the monosaccharide composition. There was no significant signal at 90 ppm, indicating that it did not contain a furan ring configuration [48]. Among them, the carbon resonance peaks between 63.36 and 76.70 ppm were assigned to C2, C3, C4 and C5 on the unsubstituted sugar ring, respectively. The carbon resonance peak at 60.29 ppm was the sugar chain C6 signal peak [49]. Therefore, by the combined ^1^H NMR and ^13^C NMR analysis of the YM25079 EPS, it was hypothesized that the EPS might be composed of α-(1→6)-linked D-glucopyranose units with α-(1→3) branching.

### 3.11. Rheological Measurements

**Figure 7:**
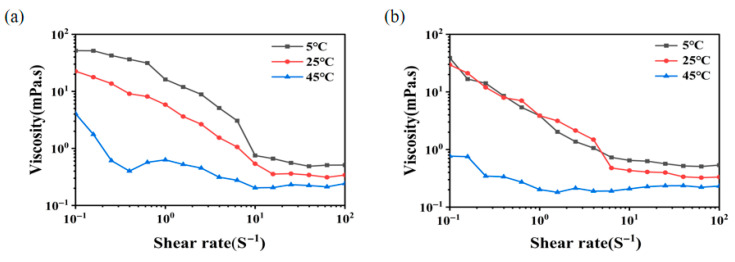
Effect of temperature on the apparent viscosity of EPS Y-1 (**a**) and EPS Y-2 (**b**).

Effects of temperature on the apparent viscosity of EPS Y-1 and EPS Y-2 are plotted in Figure 7. As the temperature increased from 5 °C to 45 °C, the apparent viscosity of EPS Y-1 decreased gradually. Meanwhile the apparent viscosity of EPS Y-2 had less temperature sensitivity at a certain temperature, between 5 °C and 25 °C, and decreased dramatically once the temperature increased to 45 °C. As the temperature increased, the interaction between the electrostatic groups and the resistance to flow decreased, leading to a decrease in apparent viscosity. At high temperatures, the intermolecular distance of EPS increased, resulting in enhanced thermal motion and lower apparent viscosity. At the same time, the bonding forces between macromolecules, such as electrostatic, hydrogen and hydrophobic bonds, were suppressed at high temperatures, and thus a subtractive effect on the rheological properties can be observed [50]. These results showed that the EPS produced under heat stress had better heat resistance.

### 3.12. α-Amylase Inhibitory Activities

**Figure 8:**
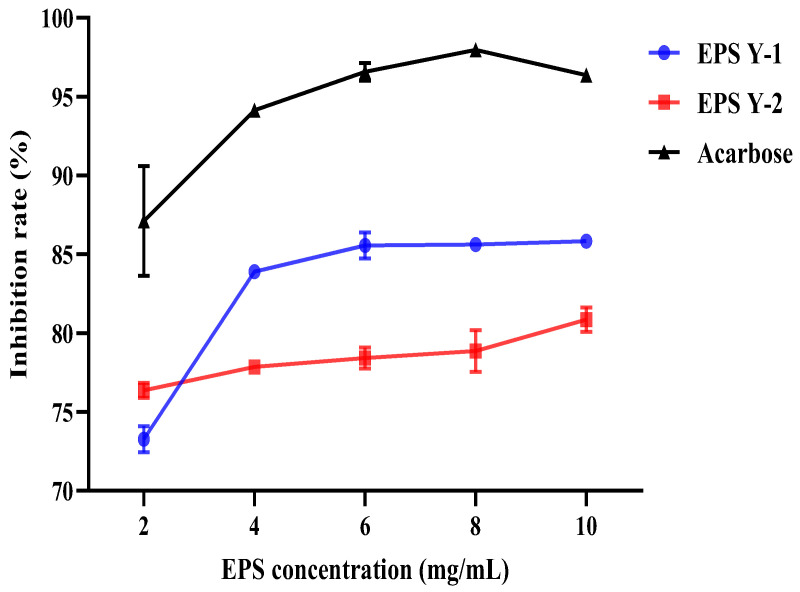
Inhibitory activity of EPS Y-1 and EPS Y-2 on α-amylase.

The inhibitory activities of EPS Y-1 and EPS Y-2 on α-amylase are shown in Figure 8. Both types of EPSs had some inhibitory effects on α-amylase, which were lower than those of the positive control acarbose. Between them, EPS Y-1 had a better inhibitory effect on α-amylase.

### 3.13. Transcriptome Analysis of the YM25079 Strain in Response to Heat Stress

To evaluate the transcriptional response of the YM25079 strain to heat stress, a transcriptome analysis was performed on two groups of YM25079 strains cultivated at 15 °C (Y79_15) and 30 °C (Y79_30) (n = 6 samples). In total, 260 million raw reads were generated; after filtering, 43 million clean reads were retained for each sample on average (Table S2). The remaining clean reads were used for assembly and 7723 unigenes were obtained (Table S3).

**Figure 9:**
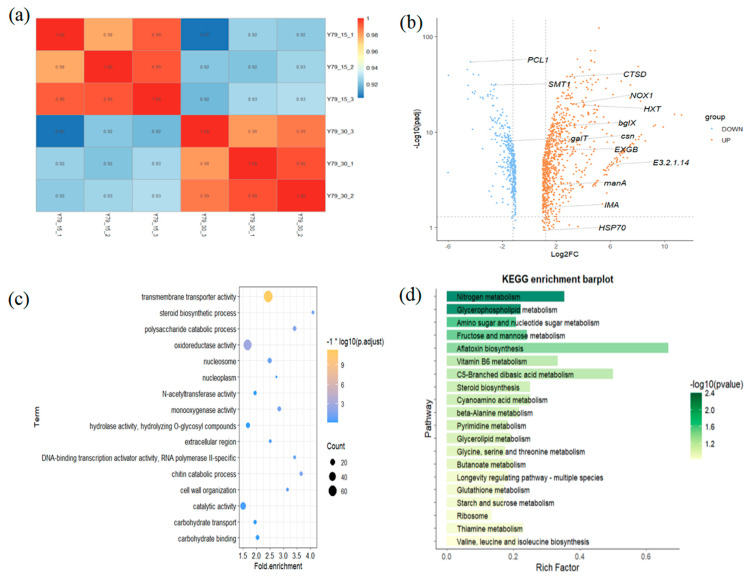
Correlation heatmap analysis of Y79_15 and Y79_30 (**a**). Volcano plots of DEGs in Y79_30 as compared to Y79_15 (**b**). GO enrichment analysis (**c**) and KEGG enrichment analysis (**d**) of DEGs.

We first used the Spearman correlation heatmap to study the differential patterns of RNA expression across different samples. The heatmap depicts that the RNA expression of YM25079 is significantly associated with heat stress (Figure 9a). Using the DESeq2 with a threshold of *p* < 0.05 and an absolute value of the log_2_ (fold change) ≥1, we identified 1282 DEGs in Y79_30 as compared to control group Y79_15. Among them, 903 (70.44%) genes were upregulated and 379 (29.56%) genes were downregulated (Figure 9b).

To gain further insights into the functions of these DEGs, we conducted GO and KEGG enrichment analyses (Tables S4 and S5). A total of 16 significantly enriched GO terms (*p* < 0.05) were found, among them, 6 GO terms remained significant after adjustment for multiple comparisons (*p*.adjust < 0.05), including “transmembrane transporter activity (GO:0022857)”, “oxidoreductase activity (GO:0016491)”, “polysaccharide catabolic process (GO:0000272)”, “chitin catabolic process (GO:0006032)”, “monooxygenase activity (GO:0004497)” and “steroid biosynthetic process (GO:0006694)”. KEGG analysis showed that seven pathways were enriched (*p* < 0.05). Figure 9d illustrates the top 20 enriched pathways, containing pathways known to play important roles in EPS synthesis such as starch and sucrose metabolism (map00500), amino sugar and nucleotide sugar metabolism (map00520) and fructose and mannose metabolism (map00051).

Table S6 lists all the differential expression analysis results as well as the specific information about the DEGs including their function annotation. Through GO and KEGG enrichment analyses, we identified a number of priority genes involved in known biological processes, such as protein processing (*CTSD*, *HSP70* and *USP12_46*), transmembrane transport (*CTSD*, *HXT*, *TPO1* and *DAL5*), starch and sucrose metabolism (*bglX*, *IMA*, *GYG1*, *PYG*), amino sugar and nucleotide sugar metabolism (*csn*, *manA*, *galT*, *E3.2.1.14*, *CHS1* and *TSTA3*), glycoside hydrolase (*bglX*, *EXGB* and *MOGS*), glycosyltransferase (*CHS1*, *ALG14*, *OGT*), response to stress (*PCL1*, *HSP70*, *NOX1*, *GST* and *KatE*) and steroid biosynthetic process (*SMT1*, *ERG26* and *ERG27*). They may play important roles in EPS metabolism under heat stress.

To confirm the accuracy of the transcriptome data, a random selection of 10 genes was used to perform RT−qPCR for quantitative analysis. All genes showed a generally consistent expression pattern between the RT−qPCR results and RNA-seq data (Figure S5), which supported that the RNA-seq dataset was suitable for further analysis.

## 4. Discussion

The study showed that the increase in exopolysaccharides of strain YM25079 was related to its stress adaptation. A study on *Rhodotorula* sp. strain CAH2 reported that EPS production was increased under abiotic stresses caused by Al, NaCl and PEG-6000 [6]. This study demonstrated heat stress could increase yeast EPS production, which has been observed in other microorganisms, such as *Lentinula edodes* [51] and Tunisian microalgae [52]. A study on Rhizobia also suggested a correlation between its EPS production and heat stress [19]. This study first investigated the effect of heat stress on the synthesis and conformational relationship of exopolysaccharides of strain YM25079. Heat stress affected the physicochemical properties and biological activities of polysaccharides by influencing the molecular weight and surface morphology of exopolysaccharides of strain YM25079. Through a series of characterization analyses of EPS Y-1 and EPS Y-2, this study showed that EPS Y-2 exhibited superior heat resistance. Therefore, under heat stress conditions, strain YM25079 secreted exopolysaccharides with better heat resistance to enhance the strain’s environmental adaptation.

To investigate the potential mechanism by which maltose promoted the synthesis of EPS by YM25079 under heat stress, the overall changes in the Y79_30 versus Y79_15 transcriptome were analyzed. It is known that heat shock response (HSR) is a highly conserved program of changes in gene expression, which results in the repression of the protein biosynthetic capacity and the induction of cytoprotective genes such as heat shock proteins (*HSPs*) against stress-induced damage. Physiological changes in the HSR includes cell cycle arrest, metabolic reprogramming, as well as altered cell wall and membrane dynamics [53]. It was worth noting that, in the Y79_30 group, the expression of a typical cyclin gene *PCL1* was significantly downregulated. This meant that the transition of cells from G1 to the S phase was inhibited [54]. At the same time, it has been shown that PCL1 affects the phosphorylation status and activity of the Glycogen Synthase gene (*GS*), in addition to controlling the cell cycle [55]. In this study, the down-regulation of PCL1 may dephosphorylate GS proteins, enhance polysaccharide synthesis activity and promote polysaccharide accumulation.

The results of KEGG map analysis and previous studies showed that the synthesis of microbial extracellular polysaccharides mainly relies on starch and sucrose metabolism (map00500), amino sugar and ribose metabolism (map00520) and glycolysis/glycolysis metabolism (map00010). Carbon sources are important nutrients and energy sources for cell growth and metabolite synthesis. It has been shown that the critical and rate-limiting step in the utilization of carbon sources is their transmembrane transport into the intracellular compartment via substrate-specific transport proteins (e.g., maltose transport proteins) [56]. In this study, the expression of the gene encoding maltose permease (*MAL31*) was up-regulated in Y79_30, and the expression of the maltase gene (*MAL32*) and the glucose transporter protein (*HXT*) gene were significantly up-regulated. It was possible that the hydrolysis of maltose activated the expression of the *HXT* gene. Thus, cells may adapt to environmental stresses through the synergistic regulation of these genes, optimizing the efficiency of carbon source utilization as well as enhancing energy supply. In addition, phosphoglucose isomerase (*PGI*) and 3-phosphoglyceraldehyde dehydrogenase (*GAPDH*) gene expressions were suppressed, suggesting that heat stress partially inhibited glycolysis in strain YM25079. Also, among the identified DEGs, many members were found to be in the glycoside hydrolases (GHs) and glycosyltransferases (GTs) families. Of note, the loss of function of glycoside hydrolase family 3 (GH3), which catalyzes glycohydrolysis and recovers key glycan components of peptidoglycan, may impair biofilm formation and extracellular polysaccharide secretion [57]. In contrast, the GTs family catalyzes the transfer of sugar groups from glycosyl donors to glycosyl acceptors to form extracellular polysaccharide repeating units and are important enzymes in polysaccharide synthesis. In addition, the phosphorylase gene (*GPH1*) was significantly down-regulated, suggesting that the degradation of polysaccharides was inhibited.

**Figure 10:**
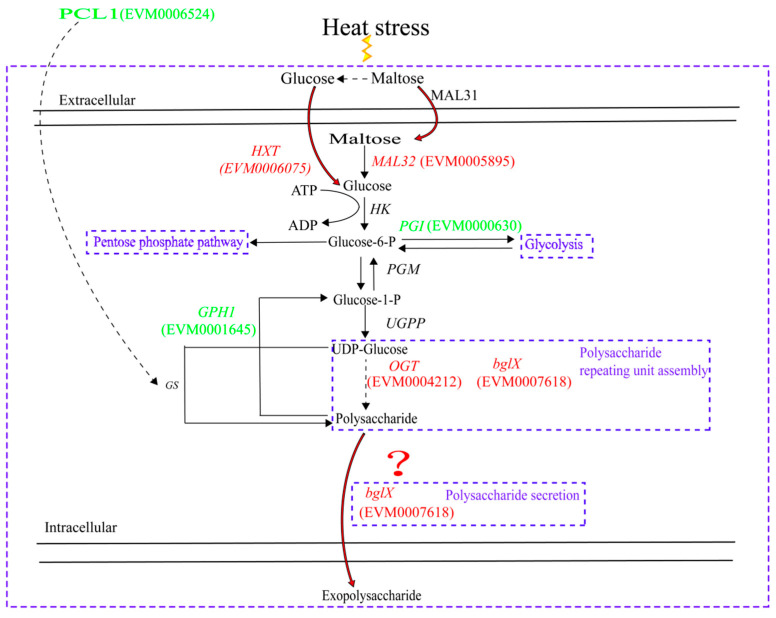
The biosynthesis of YM25079 EPS in response to heat stress. Abbreviations are as follows: MAL31 (maltose permease), *MAL32* (maltase), *HK* (hexokinase), *PGI* (phosphoglucose isomerase), *PGM* (phosphoglucomutase), *UGPP* (UDP glucose pyrophosphorylase), *OGT* (glycosyltransferase), *bglX* (glycoside hydrolase), *GPH1* (phosphorylase), *HXT* (glucose transporter protein), PCL1 (cyclin). Question mark indicates there are still many gaps in the regulation of extracellular polysaccharide secretion.

In summary, under heat stress conditions, strain YM25079 may enhance the efficiency of maltose uptake and utilization by up-regulating sugar transporter genes (*MAL31*, *HXT*) and maltose hydrolysis genes (*MAL32*), providing a rich source of substrates for polysaccharide synthesis. On the other hand, it may enhance the polysaccharide repeating unit assembly as well as the polymerization and secretion of polysaccharides by down-regulating the *GPH1* gene to reduce polysaccharide degradation and up-regulate the glycosyltransferase gene (*OGT*) and glycoside hydrolase gene (*bglX*), as shown in Figure 10. This is also a direction for us to follow to validate gene function through knockout experiments and to improve the intracellular metabolic network of the yeast in further studies.

Although this study has made some progress in exploring the EPS biosynthetic mechanism in *R. glutinis* YM25079 and their regulation of heat tolerance, several key scientific questions remain to be explored in depth. Research on the biological activities of these polysaccharides remains limited. Therefore, further investigations into the additional functions of EPS Y-1 and EPS Y-2 are needed. This would contribute to elucidating the structure–activity relationships of EPS and expand their potential applications. At the same time, due to the limited understanding of EPS biosynthesis pathways in yeast, their metabolic regulatory mechanism cannot be fully elucidated at present. In subsequent studies, further explorations towards metabolic intermediates, enzyme activities and key gene functions are needed. The metabolic regulation mechanisms of EPS biosynthesis in *R. glutinis* YM25079 under thermal stress conditions can also be investigated by integrating other omics approaches such as metabolomics and comparative genomics.

## 5. Conclusions

In this study: the production of YM25079 EPS was found to be significantly increased under heat stress. In terms of monosaccharide composition and glycosidic bond type, YM25079 EPS produced under heat stress culture was similar to that obtained from the normal environment. They were both deduced to be α-(1→6)-linked glucopyranose units with α-(1→3) branches. However, there were differences in their microstructures, and YM25079 EPS produced under heat stress had some superior properties to withstand environmental stresses, such as higher molecular weight and better thermal properties. On the other hand, the EPS obtained in the normal environment had better α-amylase inhibitory activity. In addition, transcriptomic studies revealed quite a few pathways related to the uptake and utilization of carbon, nitrogen and phosphor sources, the biosynthesis of steroid and the oxidoreductase activity, as well as potential regulatory genes implicated in the EPS biosynthesis under heat stress, which deserved to be further investigated.

## Data Availability

Transcriptome data has been deposited in NCBI with BioProject ID:PRJNA1197129.

## Supplementary Materials

The following supporting information can be downloaded at: https://www.mdpi.com/article/10.3390/jof11120883/s1, Figure S1: Purification of crude EPSs; Figure S2: UV-Vis spectrograms of EPS Y-1 and EPS Y-2 (a) and FT-IR spectrograms (b); Figure S3: ^1^H NMR spectra of EPS Y-1 (a) and EPS Y-2 (b); Figure S4: ^13^C NMR spectra of EPS Y-1 (a) and EPS Y-2 (b); Figure S5: Real-time fluorescent quantitative PCR (qPCR) versus transcriptome (RNA-seq) results validation; Table S1: Effect of different fermentation temperatures on kinetic parameters of EPS batch fermentation process; Table S2: Function annotion of unigenes; Table S3: Differential expression analysis results; Table S4: Information on the GO enrichment analysis; Table S5: Information on the KEGG enrichment analysis; Table S6: Statistical table of sequencing data.
