# Supplementary material for: Effect of Heat Stress on the Biosynthesis of Exopolysaccharides from Rhodotorula glutinis YM25079 and Its Underlying Mechanisms
Source: J Fungi (Basel). 2025 Dec 14;11(12):883. doi: 10.3390/jof11120883 (PMC12733683; doi:10.3390/jof11120883)
Supplement: Supplementary file 1 [file jof-11-00883-s001.zip › Figure S1. Purification of crude EPSs.pdf]

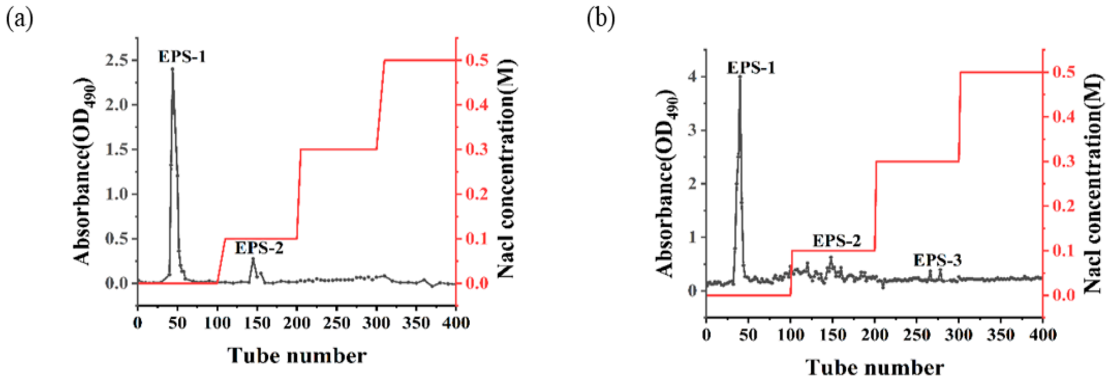

Figure S1. Eluvial curve of cellulose column chromatography for YM25079 crude EPSs produced under normal culture conditions (a) and heat stress (b).
