# Supplementary material for: Effect of Heat Stress on the Biosynthesis of Exopolysaccharides from Rhodotorula glutinis YM25079 and Its Underlying Mechanisms
Source: J Fungi (Basel). 2025 Dec 14;11(12):883. doi: 10.3390/jof11120883 (PMC12733683; doi:10.3390/jof11120883)
Supplement: Supplementary file 1 [file jof-11-00883-s001.zip › Figure S5. Real-time fluorescent quantitative PCR (qPCR) versus transcriptome (RNA-seq ) results validation.pdf]

（a）

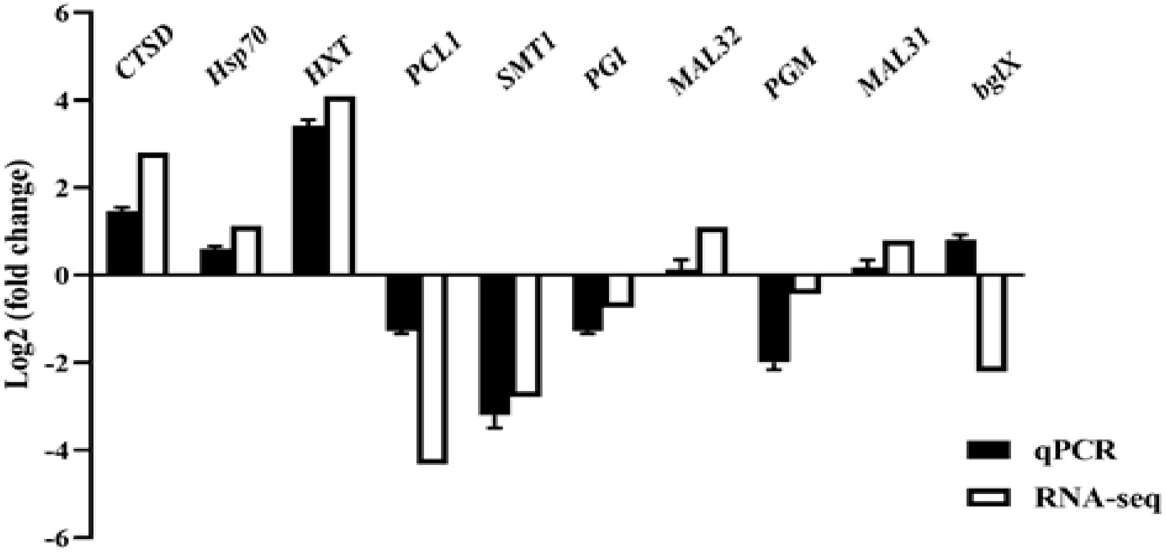

（b）

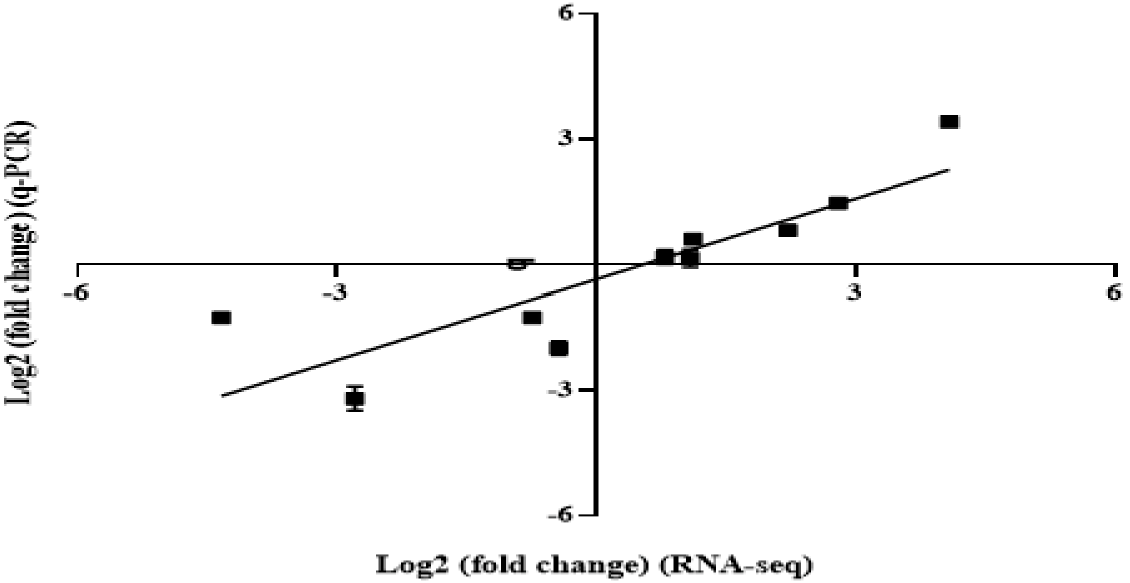

Figure S5 Real-time fluorescent quantitative PCR (qPCR) versus transcriptome (RNA-seq) results validation
