# Supplementary material for: Effect of Heat Stress on the Biosynthesis of Exopolysaccharides from Rhodotorula glutinis YM25079 and Its Underlying Mechanisms
Source: J Fungi (Basel). 2025 Dec 14;11(12):883. doi: 10.3390/jof11120883 (PMC12733683; doi:10.3390/jof11120883)
Supplement: Supplementary file 1 [file jof-11-00883-s001.zip › Table S1. Effect of different fermentation temperatures on kinetic parameters of EPS batch fermentation process.pdf]

Table S1 Effect of different fermentation temperatures on kinetic parameters of EPS batch fermentation process.

	Kinetic parameter	Fermentation conditions	
		15℃	30℃
Cell growth	X_0	0.06177	0.03828
	Xmax	2.39115	1.99883
	μ_{max}/h^{-1}	0.13286	0.33528
	R^2	0.99758	0.97863
Product synthesis	P_0	1.33330	-0.01874
	α	1.33330	2.61047
	β	0.00273	0.00146
	$\{(dp/dt)/X\}_{max}/h^{-1}$	0.17987	0.87670
	R^2	0.97325	0.90177
