# Supplementary figures and images for: Effect of Heat Stress on the Biosynthesis of Exopolysaccharides from Rhodotorula glutinis YM25079 and Its Underlying Mechanisms
Source: J Fungi (Basel). 2025 Dec 14;11(12):883. doi: 10.3390/jof11120883 (PMC12733683; doi:10.3390/jof11120883)

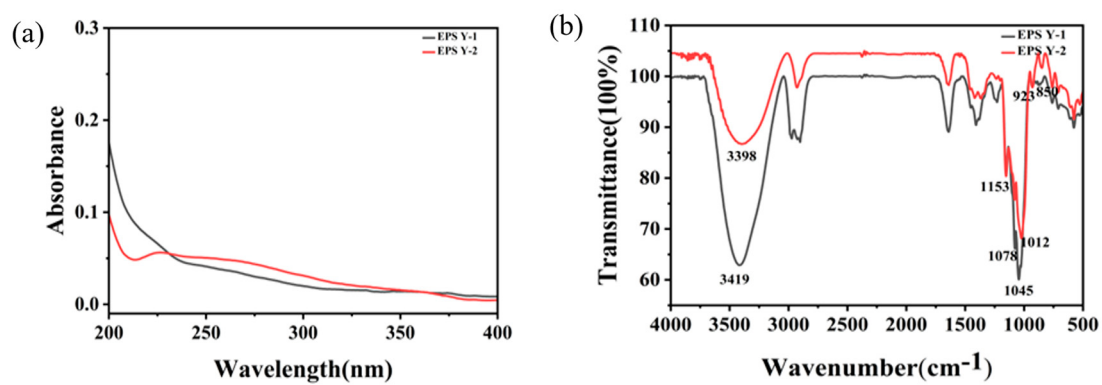

Figure S2. UV-Vis spectrograms of EPS Y-1 and EPS Y-2 (a) and FT-IR spectrograms (b)

Supplement: Supplementary file 1 [file jof-11-00883-s001.zip › Figure S2. UV-Vis spectrograms of EPS Y-1 and EPS Y-2 (a) and FT-IR spectrograms (b).pdf]

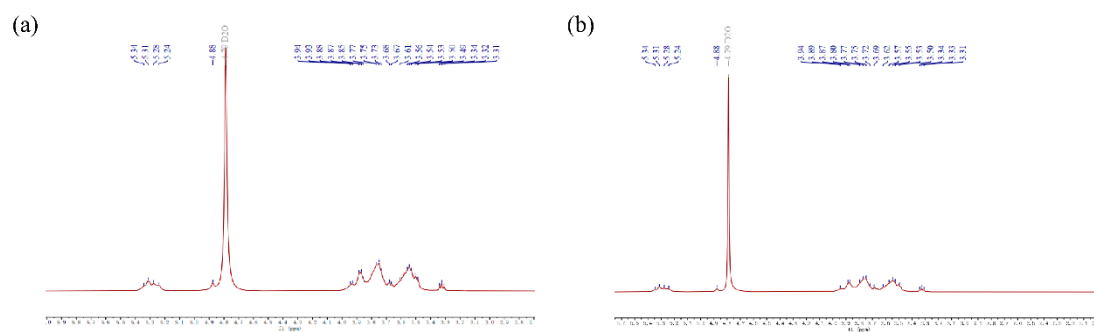

Figure S3.  $^1\text{H}$  NMR spectra of EPS Y-1 (a) and EPS Y-2 (b).

Supplement: Supplementary file 1 [file jof-11-00883-s001.zip › Figure S3. 1H NMR spectra of EPS Y-1 (a) and EPS Y-2 (b).pdf]

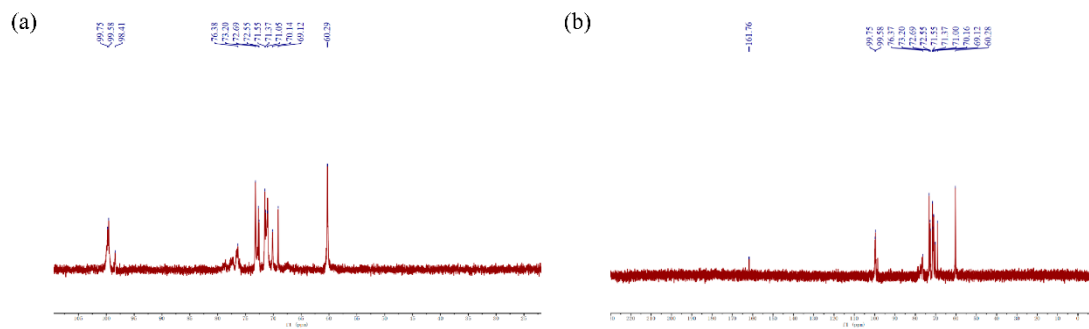

Figure S4.  $^{13}\text{C}$  NMR spectra of EPS Y-1 (a) and EPS Y-2 (b).

Supplement: Supplementary file 1 [file jof-11-00883-s001.zip › Figure S4. 13C NMR spectra of EPS Y-1 (a) and EPS Y-2 (b)..pdf]
